# Sero-Epidemiology as a Tool to Screen Populations for Exposure to *Mycobacterium ulcerans*

**DOI:** 10.1371/journal.pntd.0001460

**Published:** 2012-01-10

**Authors:** Dorothy Yeboah-Manu, Katharina Röltgen, William Opare, Kobina Asan-Ampah, Kwabena Quenin-Fosu, Adwoa Asante-Poku, Edwin Ampadu, Janet Fyfe, Kwadwo Koram, Collins Ahorlu, Gerd Pluschke

**Affiliations:** 1 Noguchi Memorial Institute for Medical Research, University of Ghana, Legon, Ghana; 2 Molecular Immunology, Swiss Tropical and Public Health Institute, Basel, Switzerland; 3 University of Basel, Basel, Switzerland; 4 National Buruli Ulcer Control Programme, Disease Control Unit - GHS, Accra, Ghana; 5 Victorian Infectious Diseases Reference Laboratory, North Melbourne, Victoria, Australia; Kwame Nkrumah University of Science and Technology (KNUST) School of Medical Sciences, Ghana

## Abstract

**Background:**

Previous analyses of sera from a limited number of Ghanaian Buruli ulcer (BU) patients, their household contacts, individuals living in BU non-endemic regions as well as European controls have indicated that antibody responses to the *M. ulcerans* 18 kDa small heat shock protein (shsp) reflect exposure to this pathogen. Here, we have investigated to what extent inhabitants of regions in Ghana regarded as non-endemic for BU develop anti-18 kDa shsp antibody titers.

**Methodology/Principal Findings:**

For this purpose we determined anti-18 kDa shsp IgG titers in sera collected from healthy inhabitants of the BU endemic Densu River Valley and the Volta Region, which was so far regarded as BU non-endemic. Significantly more sera from the Densu River Valley contained anti-18 kDa shsp IgG (32% versus 12%, respectively). However, some sera from the Volta Region also showed high titers. When interviewing these sero-responders, it was revealed that the person with the highest titer had a chronic wound, which was clinically diagnosed and laboratory reconfirmed as active BU. After identification of this BU index case, further BU cases were clinically diagnosed by the Volta Region local health authorities and laboratory reconfirmed. Interestingly, there was neither a difference in sero-prevalence nor in IS*2404* PCR positivity of environmental samples between BU endemic and non-endemic communities located in the Densu River Valley.

**Conclusions:**

These data indicate that the intensity of exposure to *M. ulcerans* in endemic and non-endemic communities along the Densu River is comparable and that currently unknown host and/or pathogen factors may determine how frequently exposure is leading to clinical disease. While even high serum titers of anti-18 kDa shsp IgG do not indicate active disease, sero-epidemiological studies can be used to identify new BU endemic areas.

## Author Summary

Sero-epidemiological analyses revealed that a higher proportion of sera from individuals living in the Buruli ulcer (BU) endemic Densu River Valley of Ghana contain *Mycobacterium ulcerans* 18 kDa small heat shock protein (shsp)-specific IgG than sera from inhabitants of the Volta Region, which was regarded so far as BU non-endemic. However, follow-up studies in the Volta Region showed that the individual with the highest anti-18 kDa shsp-specific serum IgG titer of all participants from the Volta Region had a BU lesion. Identification of more BU patients in the Volta Region by subsequent active case search demonstrated that sero-epidemiology can help identify low endemicity areas. Endemic and non-endemic communities along the Densu River Valley differed neither in sero-prevalence nor in positivity of environmental samples in PCR targeting *M. ulcerans* genomic and plasmid DNA sequences. A lower risk of developing *M. ulcerans* disease in the non-endemic communities may either be related to host factors or a lower virulence of local *M. ulcerans* strains.

## Introduction

Buruli ulcer (BU), a severe necrotizing skin disease, is caused by the environmental pathogen *Mycobacterium ulcerans* (*M. ulcerans*). Globally, it is the third most prevalent mycobacterial disease that affects immunocompetent individuals after tuberculosis and leprosy [1]. Currently more than 30 countries, mainly in the Tropics and sub-Tropics, are known to report BU cases [2]. The main countries that are severely affected lie along the Gulf of Guinea and include Ivory-Coast, Ghana, Togo, Benin and Cameroon. In the highly endemic countries BU is second after tuberculosis as the most prevalent mycobacterial disease [2], [3]. However, the global burden of BU is not clear, because efficient and comprehensive reporting systems are lacking in many of the BU endemic countries. One characteristic of BU is its focal distribution within highly endemic countries. Most cases occur in remote villages with limited access to the formal health sector, prompting affected people to seek health at traditional healers [4]. Even today, not all affected communities may be known to the National BU Control Programs. Therefore reliable tools to detect and monitor the presence of BU in communities are urgently needed.

The disease presentation, which varies between individuals, starts either as a papule, nodule, plaque or edema and if these non-ulcerative early forms are not treated, extensive tissue destruction leads to the formation of large ulcerative lesions with characteristic undermined borders. Extensive tissue destruction frequently causes disfigurement and long lasting deformities such as loss of limbs and essential organs, like the eye [5], [6]. Many features of BU such as the mode of *M. ulcerans* transmission and risk factors for an infection with the pathogen are not clearly understood. However, BU is known to occur mainly in children less than 15 years of age and affects people in wetlands and disturbed environments [3], [7]. The pathology of BU is primarily associated with the secretion of the cytocidal and immunosuppressive polyketide toxin mycolactone [8].

Current methods for a laboratory confirmation of clinical BU diagnosis include microscopic detection of acid fast bacilli (AFB), culture of *M. ulcerans*, histopathology and detection of *M. ulcerans* DNA by PCR. Currently, PCR detection of the *M. ulcerans* specific insertion sequence IS*2404* is the gold standard for BU diagnosis [9]. Yet, PCR requires elaborate infrastructure and expertise and therefore make it out of reach for primary health care facilities in BU endemic low resource countries. Serology represents a more attractive approach for the development of a simple test format that can be applied to facilities treating BU in low resourced countries. Unfortunately, various studies have shown that serological tests targeting *M. ulcerans* antigens are not suitable to differentiate between patients and exposed but healthy individuals as both groups may exhibit serum IgG titers against these antigens [10], [11]. However, serology may be a useful tool for monitoring exposure of populations to *M. ulcerans*, although great antigenic cross reactivity between *M. ulcerans*, *M. tuberculosis*, BCG and other environmental mycobacteria complicates this approach. We previously profiled an immunodominant 18 kDa small heat shock protein (shsp) absent from *M. tuberculosis* and *M. bovis* as a suitable target antigen for sero-epidemiological studies. In spite of the presence of sequence homologues in *M. leprae* and *M. avium*, Western blot analyses, using a limited number of sera indicated that this protein can be used to distinguish between *M. ulcerans* exposed and non-exposed populations [10]. Here we have extended these studies with larger sets of sera. These sero-epidemiological studies identified a BU index case in a region of Ghana that was regarded, so far, as BU non-endemic.

## Materials and Methods

### Ethics statement

Ethical clearance for the study was obtained from the institutional review board of the Noguchi Memorial Institute for Medical Research (Federal-wide Assurance number FWA00001824). Written informed consent was obtained from all individuals involved in the study. Parents or guardians provided written consent on behalf of all child participants.

### Study area

One part of this study was conducted in five districts of the Eastern Region including East-Akim (EA), New-Juaben (NJ), Suhum-Kraboa-Coaltar (SHC), Akwapim South (AS) and Akwapim North (AN) as well as two districts of the Greater-Accra Region comprising Ga-West (GW) and Ga-South (GS). While EA and NJ report no BU cases and AN only occasionally, the remaining four districts have communities that report BU regularly to the NBUCP. GW reports the highest number of cases with an annual average number of 100 new cases, followed by AS, GS and the SKC. This study focused on selected communities within these districts, which are all located along the Densu River.

The other part of this study was carried out in three communities of the Volta Region, namely Torgorme, Gblornu and Kasa. These communities are situated along the banks of River Volta in the North Tongu district of the Volta Region, which was so far regarded as BU non-endemic. Torgorme, Gblornu and Kasa have an estimated population of about a 1700, 350 and 160, respectively.

### Confirmation of BU endemicity in communities along the Densu River

Initial community entry was done by first meeting community opinion leaders, which included the disease control officer responsible for the area, the assembly man and chiefs in order to explain the importance of the activity and to solicit their cooperation. A rough sketch and count of houses along the length and breadth of the community was carried out by walking through the community in order to estimate an approximate number of houses to be surveyed. The area was then divided into two blocks, with one research team being responsible for one block. Each habitable structure within a block was then numbered serially. A house to house survey was carried out and interviews involving the head of a house were done. A data collection chart was used to collect information on the number of people in the house, healed and active BU cases and if active cases were found, samples were collected for confirmation of BU. Collected data of the BU patients included age and sex, when the disease was contracted and GPS coordinates of their houses.

### Participants and sampling for serological analysis

Two milliliters of blood were collected into vacutainer tubes (BD) from participants of ten different villages within a 5 km radius along the Densu River; six and four of the communities were confirmed as BU endemic and non-endemic, respectively, using active search and mapping activities as described above. The endemic communities were: Kojo-Ashong and Otuaplem in the GW, Kwame Anum and Ayitey Kortor in the GS, Sakyikrom and Tetteh Kofi in the AS district, respectively. The non-endemic communities were Obuotumpan and Abotanso in the NJ and Abesim Yeboah and Ntabea in the EA district. The study participants aged between 5 and 90 years recruited from these communities were individuals with no history of BU. 188 participants were from the non-endemic villages (94 each females and males); age range 5–84 years, arithmetic mean of 28.6 years, median 19 years and mode of 15. 294 participants were from endemic villages (139 and 155 were male and females, respectively); age range 5–90 years, mean age of 26.8 years, median 20 and mode 12 years.

In addition, whole blood samples were also collected from 99 community members in three villages along the Volta River in the Volta Region which has so far been considered one of the non-endemic regions in Ghana. The three communities Torgorme, Gblornu and Kasa were selected as having never reported leprosy in the past five years according to data of the North Tongu District Directorate of Health Services.

Blood samples were transported immediately at ambient temperature to the laboratory for separation of serum by centrifugation at 2,000 g for 10 mins to remove the clot. Sera were stored at −80°C until analysis.

### Western blot analysis

25 µg of recombinant *M. ulcerans* 18 kDa shsp protein was separated on NuPAGE® Novex 4–12% Bis-Tris ZOOM™ Gels, 1.0 mm IPG well (Invitrogen) using NuPAGE ® MES SDS Running Buffer (Invitrogen) under reducing conditions and transferred to nitrocellulose membranes. Membranes were blocked with 5% skim milk in phosphate-buffered saline (PBS), 0.1%Tween 20 (PBS-T) and cut into strips. Protein strips were incubated with serum samples at a 1∶500 dilution in PBS-T for 1.5 hrs. Strips were washed with 0.3 M PBS, 1% Tween 20 and incubated with alkaline phosphatase-conjugated AffiniPure F(ab′)_2_ fragment goat anti-human immunoglobulin G (IgG, Milian). Nitro blue tetrazolium (NBT) and 5-bromo-4-chloro-3-indolyl phosphate (BCIP) (BioRad) were used for color development.

### ELISA

96-well Nunc-Immuno Maxisorp plates (Thermo Scientific) were coated with 0.5 µg recombinant 18 kDa shsp per well in 100 µl PBS. Plates were incubated at 4°C overnight. Plates were washed with dH_2_O, 2.5% Tween 20 (dH_2_O-T) and blocked for 1 h with 200 µl blocking buffer (5% skim milk in PBS) at 37°C. Serial 2-fold dilutions of serum from 1∶100 to 1∶12800 in 50 µl blocking buffer per well were incubated for 1.5 hrs at 37°C. The wells were washed with dH_2_O-T. 50 µl of 1∶6000 diluted goat anti-human IgG (γ-chain specific) coupled to horseradish Peroxidase (HRP, SouthernBiotech) was added to each well and incubated for 1 h at room temperature. After the last washing step with dH_2_O-T, 100 µl TMB Microwell Peroxidase Substrate (KPL) was added. The reaction was stopped after 5 min. The absorbance was measured using an ELISA plate reader (Sunrise, Tecan) at 450 nm.

Each ELISA plate contained two-fold dilutions of a negative control comprising a pool of 5 negative sera from people living in BU non-endemic communities in Ghana and a positive control consisting of 5 medium positive sera from people living in BU endemic areas. The cut-off value for positivity was considered to be the mean optical density (OD) of negative and positive control at a 1∶100 serum pool dilution. Statistically, data were analyzed using GraphPad Prism version 5.0 (GraphPad Software, San Diego California USA). The nonparametric Kruskal-Wallis test with Dunn's post-test was used to compare OD values for the different groups.

### PCR analysis of environmental samples

Sampling was done from aquatic environments and from communities. Water, insects, fish, snails, dominant vegetation (both dead and living) and soil were collected randomly from the ground and edges of rivers at various locations in both endemic and non-endemic communities. Soil, vegetation and animal droppings were collected from various locations within both endemic and non-endemic communities. All collected samples were transported on the same day to the laboratory, stored at 4°C and analyzed within a week of collection.

DNA was extracted from about 200 mg portions of all the environmental samples using the FastDNA Spin kit for soil (MP Biomedical) according to the manufacturer's instruction. For insect samples additionally glass beads were added to the lysing matrix and the breaking step with the Fast Prep instrument was substituted by heating specimens at 95°C for twenty minutes followed by vortexing full speed for two minutes. The extracted DNA was stored at −20°C until analysis by real-time PCR.

TaqMan real-time PCR was performed using primers and procedures as previously described with some modifications in reaction conditions [12]. The primers and TaqMan MGB probes detecting IS*2404*, IS*2606* and the ketoreductase (KR) domain were obtained from Applied Biosystems (Foster City, CA, USA). IS*2404* real-time PCR mixtures contained 1X Qiagen master mix (containing HotstarTaq plus DNA polymerase, dNTP mix and PCR buffer) 1 µl of extracted template DNA, 0.5 µM concentrations of each primer and 0.2 µM probe, 1× TaqMan exogenous internal positive control (IPC) and probe reagents (Applied Biosystems), in a total volume of 20 µl. Amplification and detection were performed with the Rotor-Gene Q (Qiagen) using the following program: 1 cycle of 95°C for 5 min, 40 cycles of 95°C for 15 s and 60°C for 15 s. Each PCR run contained 2 non-template controls and an IS*2404* positive control. Analysis for IS*2606* and KR was in a multiplex PCR using KR and IS*2606* probes with FAM and VIC fluorescence labels respectively and reaction conditions as above.

### Data analysis

Initial BU survey results were entered in Microsoft Access and exported for integration using Quantum Geographic Information System (GIS) for analyses. Google Earth aerial images of communities were obtained, geo-referenced and linked to ground contours, features and other characteristics. The prevalence of BU was calculated by counting all individuals in the community with a classical BU scar, together with those with laboratory confirmed active disease, divided by the total number of persons examined within a community. The rate was expressed as a percentage.

## Results

### *M. ulcerans* 18 kDa shsp specific serum IgG responses in individuals living in the Volta region of Ghana and identification of a BU index case in the region

**Figure 1:**
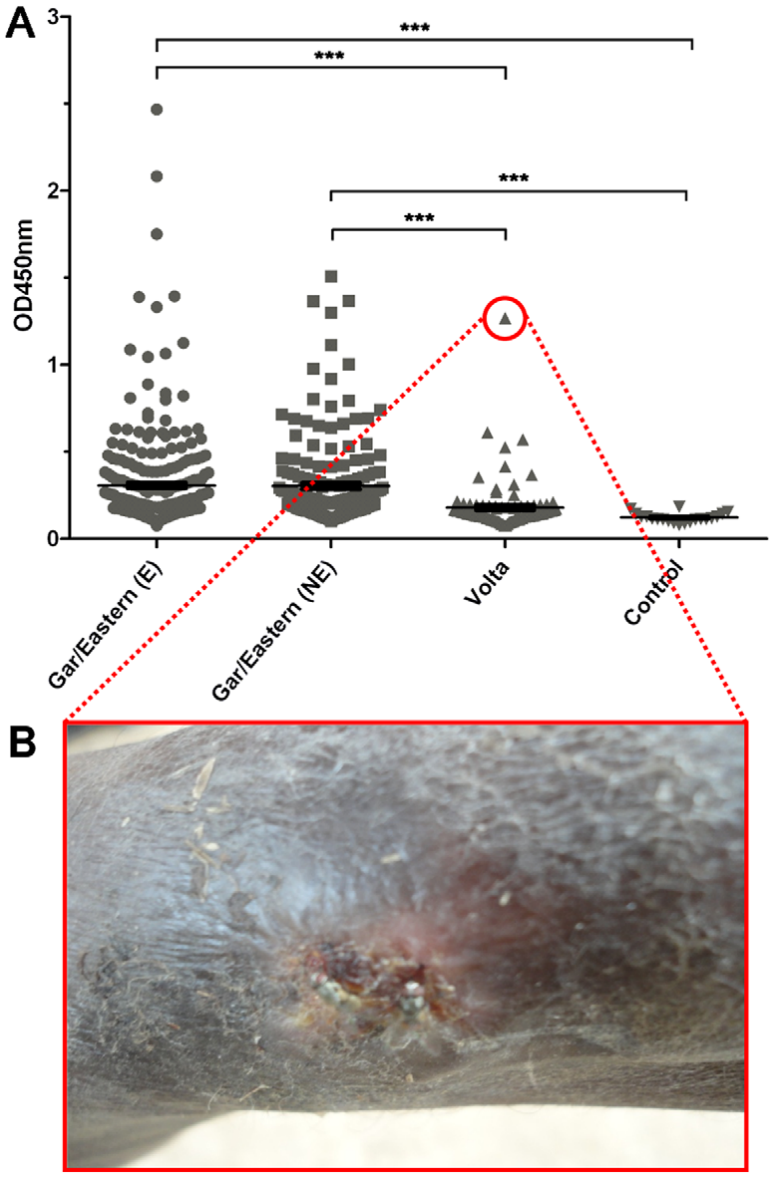
Anti-*M. ulcerans* 18 kDa shsp IgG titers. **A** OD values of sera from individuals living in BU endemic (E) and non-endemic (NE) communities along the Densu River (Gar/Eastern) or in the Volta Region (Volta) of Ghana obtained in an 18 kDa shsp ELISA for a 1∶100 serum dilution are shown for individual sera. Sera from Europeans without travel history to Africa served as controls. Statistical differences between groups were calculated by the Kruskal-Wallis test with Dunn's post test (*** = p<0.001). **B** Laboratory confirmed BU lesion of a sero-positive participant from the Volta region.

We determined *M. ulcerans* 18 kDa shsp-specific serum IgG titers in 482 sera from people living in the BU endemic Densu River Valley in the Gar and Eastern Region, 99 sera from people living in the BU non-endemic Volta Region and 20 sera from European controls without travel history to Africa (figure 1A). Based on the defined ELISA OD cut-off values, a sero-positivity rate of 32% was observed for the sera from the Densu River Valley. The sero-positivity rate of people living in the Volta Region (12%), as well as the mean ELISA readouts obtained with their sera were significantly lower (p<0.001). None of the sera from European controls exhibited a significant titer (figure 1A). Sero-positive individuals from the Volta region were re-visited and interviewed. It was determined that all of them have lived entirely or at least for most of their life in their home communities in the Volta Region. One of the sero-positive participants from the village Torgorme reported at the interview to have a non-healing chronic wound on the leg (figure 1B). The wound was clinically diagnosed by an experienced physician as BU and clinical diagnosis was laboratory reconfirmed by positive IS*2404* PCR of swab specimens. The serum of this reconfirmed BU patient had the highest anti-18 kDa shsp-specific serum IgG titer of all participants from the Volta Region tested (figure 1A).

Following the identification of this index case, the health directorate of the Volta Region sent us specimens from eleven other individuals with suspected BU lesions. Six of these, were reconfirmed as IS*2404* PCR-positive BU by our laboratory at the Noguchi Memorial Institute for Medical Research, which is one of the BU reference laboratories in Ghana. While sera of two laboratory confirmed BU patients contained anti-18 kDa shsp IgG, four patients were sero-negative.

### Comparison of anti-18 kDa shsp IgG sero-positivity in BU endemic and non-endemic communities along the Densu River

**Figure 2:**
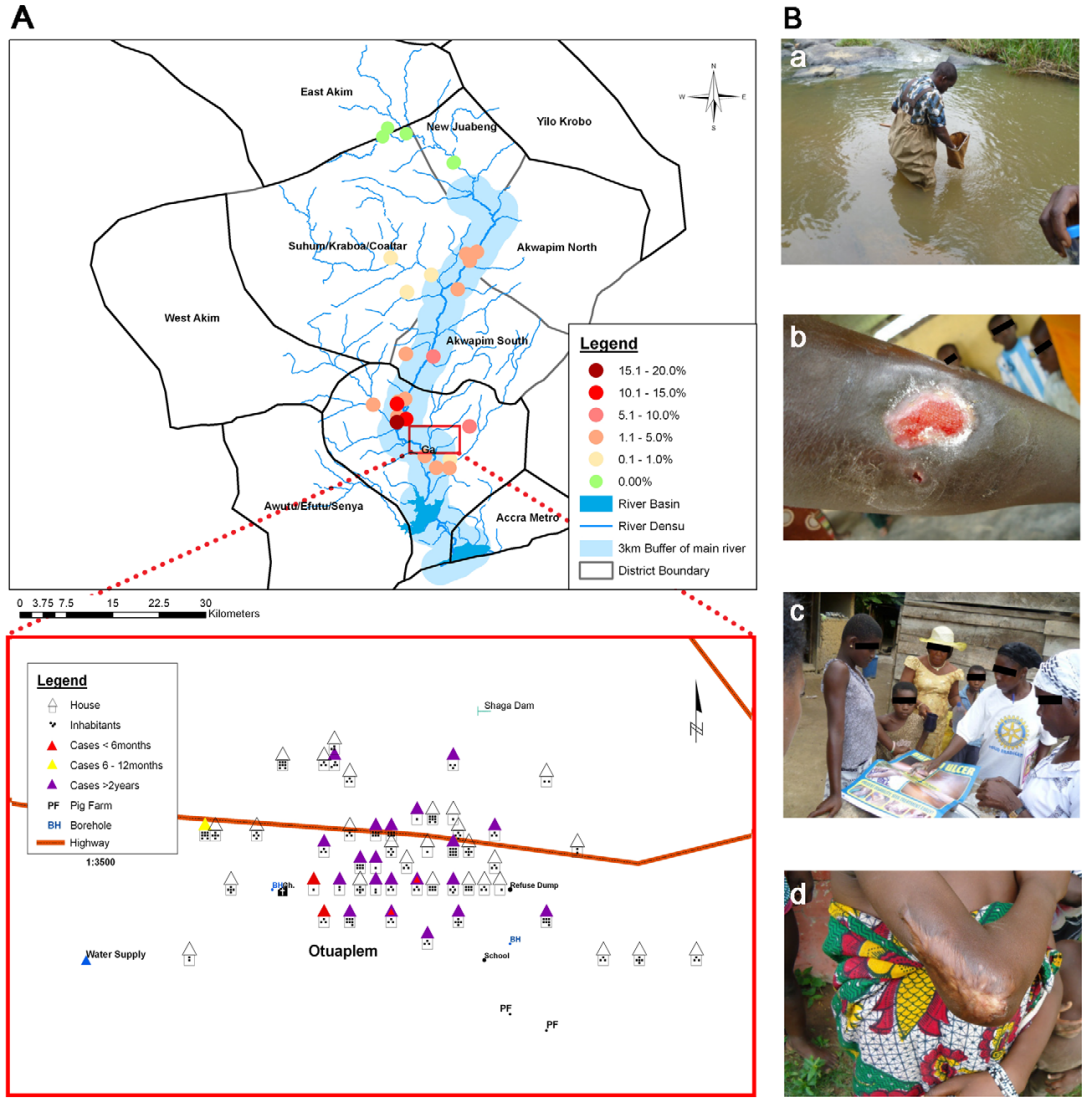
Prevalence of BU and sample collection procedure. **A** Map showing the prevalence of BU in selected communities along the Densu River. The prevalence rate was calculated by adding the number of individuals both with active or healed BU lesions divided by the total population number of the community. Exemplarily, the housing and population census is illustrated for the village Otuaplem. **B** Photographs illustrating environmental sampling (a), reconnaissance visit in the communities (b), an active BU lesion found in a community during active search (c) as well as a healed BU lesion also identified in a community (d).

Active case search surveys were performed to determine the prevalence of BU along the Densu River (figure 2). The average prevalence of BU in endemic communities with 3 km buffer was 3.4%. While in some communities upstream no BU cases were found, the disease burden increases as the River runs downstream (figure 2A). Of the ten communities included in the sero-epidemiological study, four (Ntabea, Abesim-Yeboah, Obotanso and Obuotupan) were confirmed as non-endemic, as both the passive surveillance by the National BU control program and our active case search identified neither healed nor active cases. The total prevalence rate, including both healed and active cases, of the six endemic communities ranged from 1% to 19% with Tetteh Kofi, Otuaplem and Sode having the highest rates (4.8%, 14.9% and 19.1%, respectively). The prevalence of active cases ranged from <1% to 2.4%, with Sode also having the highest active case prevalence rate.

**Figure 3:**
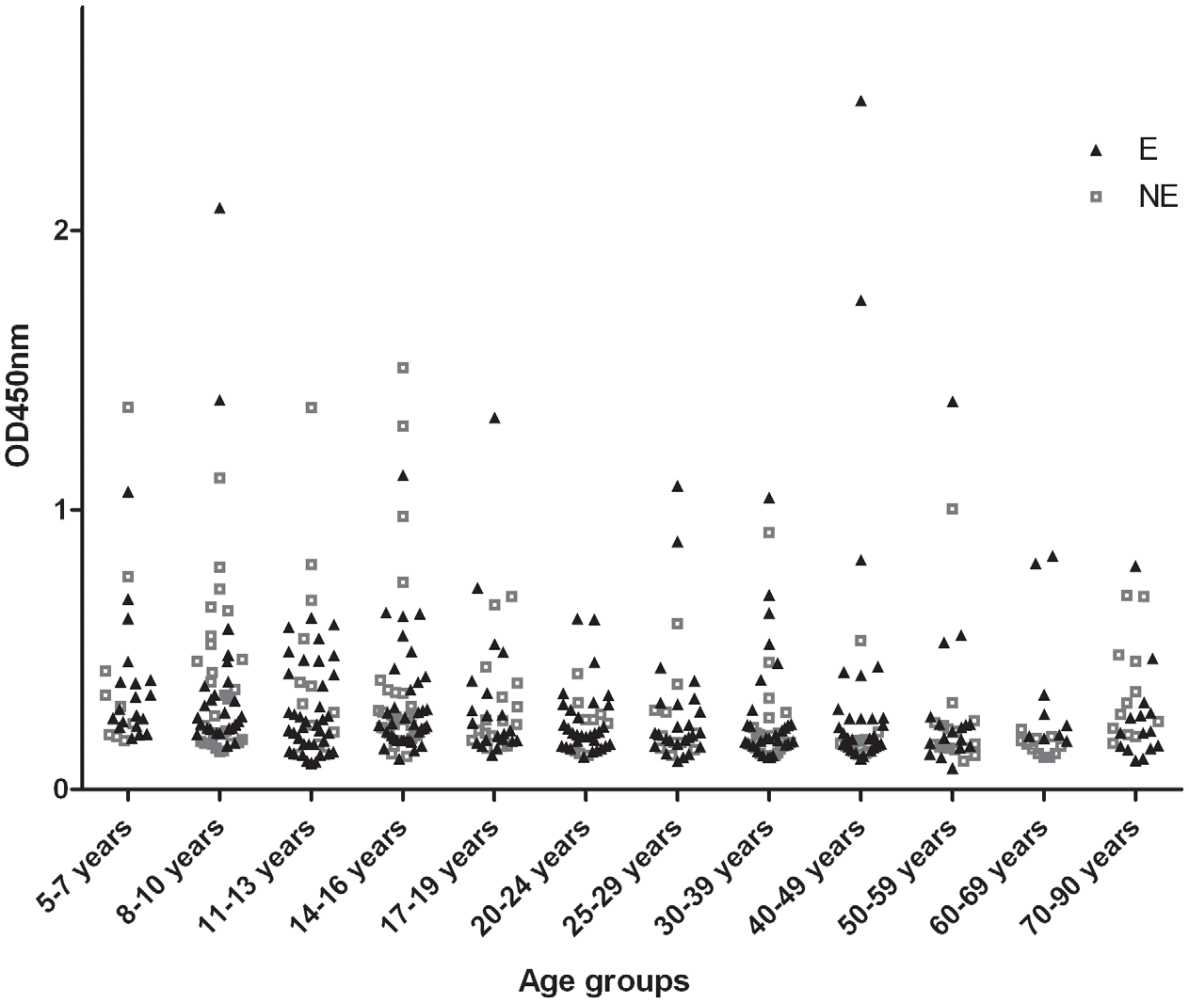
Age distribution of anti-18 kDa shsp IgG titers. OD values of sera from individuals living in BU endemic (E) and non-endemic (NE) communities along the Densu River obtained in an 18 kDa shsp ELISA for a 1∶100 dilution are shown for individual sera.

When 18 kDa shsp-specific serum IgG titers of 295 sera from BU endemic and of 187 sera from non-endemic communities were analyzed by ELISA (figure 1A), comparable sero-positivity rates (33% versus 31%, respectively) were found. ELISA results were reconfirmed by Western blot analysis with a randomly chosen subset of sera. There was good agreement between Western blot band intensities and ELISA titers with a few discrepancies related to a higher sensitivity of the ELISA method (data not shown). Sero-responders were found in all age groups (>5 years) tested, but sero-negative individuals dominated throughout (figure 3).

### PCR analysis of environmental samples

211 environmental samples were collected randomly from both aquatic and dry land environs. The sampled BU endemic communities included Kojo-Ashong (KA), Sode (SD), Amasaman and surrounding hamlets (AS), and Kudeha and surrounding hamlets (KD) located in the GW and GS districts. Samples from non-endemic communities were collected in Abesim-Yeboah (AY), Obuotumpan (OB) and Ntabea (NB) located in the EA and NJ districts further up-stream of the Densu River (figure 2A).

*M. ulcerans* DNA in an environmental sample was confirmed by the presence of all three tested loci (IS*2404*, IS*2606* and KR) as revealed by positive results with all three PCR tests performed. In all, 19/211 (9.0%) of the samples tested were positive, including 5/19 aquatic snails, 5/28 sand samples collected from the communities, 4/30 samples from river water and river bed sand, 2/30 samples from aquatic vegetation, 1/6 sand samples collected from farms, 1/12 aquatic insects and 1/1 millipedes. As shown in Table 1 the average positivity rates for samples from endemic communities were 13.4% (7.7%, 13.3%, 14.3% and 18.5% in KA, AS, SD and KD, respectively) and 6.2% for samples from non-endemic communities (26.0%, 2% and 1.8% for NB, OB and AY, respectively).

**Table 1:** IS*2404*, IS*2606*, KR PCR positivity of environmental samples collected in communities along the Densu River.

Village	BU endemicity	BU prevalence*	PCR positivity**
Obuotupan	Non-endemic	0%	1/50 (2%)
Abesim Yeboah	Non-endemic	0%	1/56 (1.78%)
Ntabea	Non-endemic	0%	6/23 (26%)
Amasaman	Endemic	5.1%	2/15 (13.3%)
Kudeha	Endemic	3.2%	5/27 (18.51%)
Kojo Ashong	Endemic	14.3%	2/26 (7.69%)
Sode	Endemic	19.1%	2/14 (14.28%)
**Total**			**19/211 (9%)**

*Prevalence rate is given as the total prevalence comprising both active and healed lesions.

**Positivity indicates the presence of all three loci targeted by RT-PC.

## Discussion

Broad antigenic cross-reactivity between mycobacterial species represents a major challenge for the development of a serological test that is specific and sensitive enough to monitor immune responses against *M. ulcerans* in populations where exposure to *M. tuberculosis* and BCG vaccination is common. In our earlier work, we have identified the *M. ulcerans* 18 kDa shsp as an immunodominant antigen, which has no homologues in *M. tuberculosis* and *M. bovis* [10]. However, interspecies cross-reactivity of this protein with an 18 kDa protein of *M. leprae* as well as a 20 kDa protein of *M. chelonae* was detected. In the same study we evaluated the use of measuring anti-18 kDa shsp IgG titers for assessing the exposure of a population to *M. ulcerans* on the basis of a limited number of BU patients, household contacts and people living in areas where BU is not endemic [10]. Since sera from inhabitants of BU non-endemic regions showed largely no reactivity with the 18 kDa protein of *M. ulcerans*, immune responses against environmental mycobacteria, such as *M. chelonae*, do not seem to compromise the developed serological test for *M. ulcerans* exposure. Here we have extended our previous analysis by comparing sera from areas of Ghana, which rarely report leprosy cases, but differ in their reported BU endemicity.

In Ghana, a national case search performed in 1999 yielded a crude national BU prevalence rate of 20.7/100,000 and hence demonstrated that BU is the second most common mycobacteriosis in the country after tuberculosis [13]. In this study diagnosis of both active and healed lesions was based solely on clinical grounds without any microbiological confirmation. Since the creation of the national control program, 32 of the 166 nation-wide districts continuously report BU. Through this passive surveillance system, over 11,000 cases have been reported between 1993 and 2006 (http://www.who.int/mediacentre/factsheets/fs199/en/) from mainly six of the ten regions of Ghana. No BU cases have been reported from the Volta, Northern, Upper East and West regions, giving the impression that those four regions do not harbor BU cases and therefore are non-endemic. However, in our analysis of sera from the Volta Region, a relatively small, but significant number of serum samples contained anti-18 kDa shsp IgG. Follow-up visits and interviews revealed that one of the sero-positive individuals had a chronic wound which was subsequently laboratory confirmed as BU [14]. After identification of this index case, additional laboratory confirmed BU cases were found by active case search in the Volta Region. In our previous analyses [10], only part of the sera from laboratory reconfirmed BU patients were tested postitive for anti-18 kDa shsp IgG. In accordance with these findings, not all of the BU patient sera from the Volta region were sero-positive. These data clearly show that anti-18 kDa shsp IgG titers are no indication for active disease. A large epidemiological survey is now required to determine the prevalence of BU over the entire Volta Region. Until today no serological test allows for a distinction of BU patients and healthy individuals, which are exposed to *M. ulcerans*. However, our results demonstrate that sero-epidemiological studies can be used to complement active case search in regions, where data about the BU prevalence are lacking. Future longitudinal sero-epidemiological studies are planned in order to monitor the exposure of certain populations to *M. ulcerans* over a longer period of time. At this stage we cannot conclude how timing and frequency of exposure influences antibody titers against the pathogen.

The prevalence of 18 kDa shsp sero-positive individuals within populations along the Densu River was >30%. This confirms our earlier conclusion that a large proportion of healthy individuals living in endemic communities who have responded immunologically to *M. ulcerans* exposure do not develop overt disease. While the percentage of 18 kDa shsp sero-positive individuals was higher compared to that found using a Burulin skin test in healthy controls, it is comparable to that obtained for the serologic response to *M. ulcerans* culture filtrate [15],[16]. Diverse outcome of infection with the causative agents of the main mycobacterial diseases such as tuberculosis seems to be a common feature of their natural history. Not all exposed individuals show immunological evidence of infection and of those who get infected by *M. tuberculosis* estimations indicate that only 10% will ever develop overt disease [17], [18]. Manifestation of the disease ranges from self-limited pulmonary infection to localized extra-pulmonary infection and disseminated disease [19]. Factors accounting for the diversity in outcomes are not entirely known, but may relate to both host and pathogen factors. Even though clinical *M. ulcerans* isolates from Africa are clonally related and genetically largely monomorphic [20]–[27], differences in virulence among African *M. ulcerans* strains cannot be ruled out completely. Hence, the percentage of *M. ulcerans* infected individuals who proceed to develop BU remains to be established. BU is known to develop in all age groups with a nearly equal gender distribution but most cases occur in children 15 years of age or younger [28]. In our study we found anti-18 kDa shsp sero-responders in all age groups (>5 years) analyzed. A future cohort study with infants could provide important insight, at which age these immune responses start to emerge.

Both in endemic and non-endemic villages of the Densu River Valley we found *M. ulcerans* PCR positive environmental samples. This is indicative for the presence of *M. ulcerans* or of closely related environmental bacteria all along the Densu River. Our findings are consistent with earlier findings of Williamson et al. [29] in the same region. Since the mode of *M. ulcerans* transmission and risk factors for the exposure to the pathogen are still not entirely elucidated, it is not clear, whether the types of environmental samples that were PCR positive have direct relevance for infection with *M. ulcerans*. Hence methods for the routine isolation and characterization of *M. ulcerans* from the environment need to be developed.

Hypotheses on risk factors and the mode of infection with *M. ulcerans* include contamination of wounds from an environmental reservoir, inhalation of vaporized contaminated water and inoculation by insects [30]–[32]. Our molecular epidemiological studies have recently demonstrated a focal transmission pattern for *M. ulcerans* [27]. This may help to explain one of the mysteries of BU transmission, the close proximity of endemic and non-endemic villages. As indicated in figure 2, while *M. ulcerans* is endemic in some villages within the Suhum-Kraboa-Coaltar district, through active case search we did not find any case (both healed and active) in neighboring districts located at the upper part of the river, such as East-Akim and the New-Juaben. In contrast, communities of the four districts, which are situated downstream (Akwapim South, Akwapim North, Ga-West and Ga-South) regularly report BU cases. BU endemic and non-endemic communities along the Densu river differ in terms of their vegetation. Upstream, within the wet semi-equatorial zone, the vegetation is predominantly moist semi-deciduous rain forest, which gradually changes downstream into a short stretch of Guinea Savannah around Nsawam and ends with coastal scrub and savannah grassland in the Ga districts. In addition, there is a variation in the features of the Densu River, which takes its source from the Atewa Forest Range near Kibi and flows for 116 km into the Weija Water Reservoir before entering the Gulf of Guinea through the Densu Delta Ramsar site. While upstream the river flows fast, has clear water and the river bed consists of rocky stones, downstream the river flows sluggishly, has a muddy river bed, and the water is turbid. We did not find significant differences in anti-18 kDa shsp IgG seropositivity rate or titers between people living in communities in the Densu River Valley that were classified based on active case search as BU endemic or non-endemic. These findings could imply at least one of the following: 1) people in the non-endemic communities in the upper Densu River Valley may be exposed to *M. ulcerans* lineages with low virulence; 2) currently unknown host genetic, behavioral or socio-economic factors trigger the development of subclinical *M. ulcerans* infection to clinical disease; 3) in the non-endemic communities 18 kDa shsp binding antibodies are triggered by subclinical infections with environmental mycobacteria harboring antigens that are cross-reactive with the 18 kDa shsp [10].
